# The possible and intriguing relationship between bullous pemphigoid and melanoma: speculations on significance and clinical relevance

**DOI:** 10.3389/fimmu.2024.1416473

**Published:** 2024-08-29

**Authors:** Filomena Russo, Anna Pira, Feliciana Mariotti, Federica Papaccio, Anna Rita Giampetruzzi, Barbara Bellei, Giovanni Di Zenzo

**Affiliations:** ^1^ Dermatological Department, Istituto Dermopatico dell’Immacolata (IDI)-Scientific Institute for Research, Hospitalization and Healthcare (IRCCS), Rome, Italy; ^2^ Molecular and Cell Biology Laboratory, Istituto Dermopatico dell’Immacolata (IDI)-Scientific Institute for Research, Hospitalization and Healthcare (IRCCS), Rome, Italy; ^3^ Laboratory of Cutaneous Physiopathology and Integrated Center for Metabolomics Research, San Gallicano Dermatological Institute, Scientific Institute for Research, Hospitalization and Healthcare (IRCCS), Rome, Italy

**Keywords:** bullous pemphigoid, melanoma, immune-checkpoint inhibitors, BP180, BP230

## Abstract

Bullous pemphigoid (BP) is the most common autoimmune bullous disease: it most commonly affects individuals over 70 years old and impacts severely on their quality of life. BP represents a paradigm for an organ-specific autoimmune disease and is characterized by circulating IgG autoantibodies to hemidesmosomal components: BP180 and BP230. While the crucial role of these autoantibodies in triggering BP inflammatory cascade is fully acknowledged, many ancillary etiological mechanisms need to be elucidated yet. Cutaneous melanoma is due to a malignant transformation of skin melanocytes, that produce and distribute pigments to surrounding keratinocytes. Melanoma is the most fatal skin cancer because of its increasing incidence and its propensity to metastasize. Several data such as: i) reported cases of concomitant melanoma and BP; ii) results from association studies; iii) BP onset following immune check-point inhibitors therapy; iv) expression of BP antigens in transformed melanocytes; and vi) circulating autoantibodies to BP antigens in melanoma patients suggest an intriguing, although unproven, possible association between melanoma and BP. However, a possible causative link is still debated and the putative pathogenetic mechanism underlying this association is unclear. This review aims to describe and discuss the possible relationship between BP and melanoma and give an overview of the speculations for or against this association. Of note, if demonstrated, this association could unwrap considerations of clinical relevance that represent new research frontiers.

## Introduction

Bullous pemphigoid (BP) is the most common autoimmune bullous disease, which typically presents with tense, itchy skin blisters affecting predominantly the inner parts of the limbs and the trunk. In up to 20% of cases, the onset of tense blistering lesions may occur after a non-bullous phase characterized by eczematous, urticaria-like, or nodular lesions (1, 2). In atypical cases, bullous lesions may be absent: these cases require a high degree of clinical suspicion and may be associated with diagnostic delay (3). BP is usually a chronic disease, with spontaneous exacerbations and remissions, that most commonly affects individuals over 70 years old, impacting severely on their quality of life. It represents a paradigm for an organ-specific autoimmune disease and is characterized by circulating IgG autoantibodies (autoAbs) to BP180 and BP230, two molecular components of the hemidesmosomes promoting dermo-epidermal cohesion. While the crucial role of these autoAbs in triggering BP inflammatory cascade is fully acknowledged, many ancillary etiological mechanisms need to be elucidated yet (1). Although BP is a rare disease, its incidence, ranging from 2.4 to 23 cases per million in the general population, is significantly raising, showing a 1.9- to 4.3-fold increase over the last two decades (4). This phenomenon could be due to the aging population, easier access to novel serological diagnostic approaches, the increasing knowledge on atypical and non-bullous forms of BP, the association with neurological disorders whose prevalence is growing, and a wide use of its triggering drugs, such as dipeptidyl peptidase IV inhibitors and immune checkpoint inhibitors (ICIs) (5, 6). BP management involves hospitalization for severe cases and the administration of high potency topical and systemic corticosteroids.

Cutaneous melanoma is a malignant transformation of skin melanocytes, the neural crest-derived cells that produce and distribute pigments to surrounding keratinocytes. Melanoma is the most fatal skin cancer because of its increasing incidence and its propensity to metastasize (7). Exposure to UV radiation is the main risk factor for melanoma, as it leads to carcinogenic mutations and suppresses some properties of the immune system (8). Melanin is a broadband UV absorbent, as well as an antioxidant and radical scavenging molecule, and skin pigmentation is the most important photoprotective factor against melanoma. As a result, a large geographical disparity exists, due to different distribution of skin phototypes and sun exposure (9). The highest incidence rate is observed in countries of fair-skinned populations with a prevalence of individuals with light-colored hair and eyes (36 per 100000 person-years in Australia) while the lower incidence corresponds to Africa and Asia (less than 1 per 100000 person-years) (10, 11).

First-line treatment for primary melanoma is surgical excision, followed in selected cases by radiotherapy or systemic therapy and, eventually, additional surgery if disease staging supports these treatments. Systemic therapy for melanoma includes chemotherapy, ICIs and target therapies. Melanoma is considered a highly “immunogenic” tumor, eliciting a powerful immune response, and immunotherapies have significantly improved treatment outcomes (12). However, the induced alteration of the immune system might cause various cutaneous adverse manifestations, including eczema, vitiligo, psoriasis, and BP (13).

Several data suggest a possible association between melanoma and BP, but the causative link is still debated and the putative pathogenetic mechanism underlying this association is unclear. However, although unproven, the association between BP and melanoma could unwrap considerations of clinical relevance that represent new research frontiers.

## Concomitant melanoma and bullous pemphigoid

Published literature reports only a few cases describing concomitant BP and melanoma, dating back to 1961, when Marks et al. reported the case of a patient who achieved BP partial remission for years after tumor excision, and experienced a flare-up when she developed a large, tender lymph node with histological signs of recurrence of melanoma. The lymph node was excised, and the patient was able to control BP lesions on minimal corticosteroid dosage (14). More recently, two more cases reported patients who developed BP and were concomitantly diagnosed with malignant melanoma. Parimi and coworkers published the case of a man with newly-diagnosed BP in whom nail dystrophy and darkening of the nail bed on his right toe was noted: according to the patient, nail changes appeared 20 years prior and were never treated. He was diagnosed with malignant melanoma and, upon further investigation, lung metastases and lymph node involvement were discovered (15). Another case developed BP with a simultaneous diagnosis of melanoma. The patient achieved control of disease activity within 2 weeks after tumor excision, without corticosteroid therapy (16). Moreover, Beck et al. reported BP onset after recurrent subungual melanoma and distant metastases in a patient with previous history of melanoma (17). It is interesting to note that, in described cases, the course of BP seems to follow that of melanoma: BP onset and flare-ups after the appearance of primary tumor or metastases, together with skin lesions improvement after resection of the initial melanoma or metastatic lymph nodes, may underline a link between these diseases.

## Association between melanoma and bullous pemphigoid: database studies

Several studies investigated the association between BP and cancer, with controversial results, and to date, only a few studies investigate its association with melanoma. Recently, Kridin et al. attempted to evaluate the risk of developing BP in subjects with previous melanoma and found that BP patients, especially males and individuals over 80 years old, had higher prevalence rates of pre-existing melanoma than controls (1.5% vs 1.0%, respectively; p=0.004). Conversely, the risk of incident melanoma among patients with BP compared to control subjects was not statistically significant (18). These data seem to be reinforced by Baum and coworkers, who compared the rate of malignancies in 355 BP patients and the general population. In fact, while there was no difference in overall malignancy rates, BP patients had significantly higher rates of melanoma compared to the general population (10.7% vs 4.3%, respectively, p=0.0005), and in 61.5% of cases melanoma preceded BP (19).

However, conflicting data challenging the possible association between BP and melanoma were also reported. The comparison between 5739 BP patients and 17168 controls showed a 1.0 prevalence odds ratio (95% CI 0.8 – 1.3) of melanoma prior to BP diagnosis and an overall 0.7 hazard ratio (95% CI 0.5 – 1.2) for melanoma in BP patients compared to controls (20). These data agree with a Mendelian randomized analysis used to assess the causal relationship between BP and the risk of 13 types of cancer, including melanoma, that found no significant association (21).

## Bullous pemphigoid onset following immune check-point inhibitors therapy

An indirect support of the possible association between melanoma and BP is emerging evidence that treatment of melanoma with ICIs can be followed by BP onset (22). ICIs are monoclonal antibodies meant to interfere with tumor evasion mechanisms, such as the expression of inhibitory signals that prevent their detection and destruction from the immune system. ICIs target either programmed cell death-1/ligand 1 (PD-1/PD-L1) or the cytotoxic T-lymphocyte antigen-4 (CTLA-4), both involved in the negative regulation of T-cell immune function (6, 23). Although ICIs demonstrated promising anti-cancer activity in the treatment of melanoma, lung cancer and squamous cell carcinomas (SCCs) among many other malignancies, they were highly bound with immune-related adverse events, including the induction of BP (6, 23–25). In literature, the presentation of bullous disorders after ICI therapies varies from 0.3% to 1.5% of treated patients (17, 26–31) and tends to develop as a delayed immune-related adverse event, more than 4 months after therapy initiation (25). The use of monoclonal antibodies to PD-1 and PD-L1 can force the immune checkpoint of T-cells, amplifying their activity and resulting, in some cases, in a B-cell release of autoAbs to BP180 and BP230, which could lead to BP onset (6, 32). In these cases, therapeutic management of BP may negatively affect ongoing oncological treatment. Although relatively rare, this phenomenon is well documented: according to a recent review by Merli and coworkers, more than 373 cases of ICI-induced BP were published. In 85% of cases, BP followed the use of PD-1 inhibitors pembrolizumab or nivolumab; the mean time from first administration to BP onset was 26 weeks, and in 6.7% of patients it occurred after ICI discontinuation (6). The majority of patients were treated for melanoma (42%) and non-small-cell lung cancer (25%) (6): since ICI therapy is used to treat more lung cancer than melanoma (ratio=2.3) (33), the highest prevalence of BP after melanoma treatment suggests a preferential link between them. Patients with idiopathic and ICI-induced BP show some differences: the latter tend to be younger and predominantly male (34), with variable clinical features. ICI-induced BP is generally milder than the idiopathic form and less likely to present with tense bullae, with longer rash-free pruritus and significantly longer delay from symptom onset to BP diagnosis; moreover, immunopathological and serological features were confirmed in less cases (28, 35–37) and reactivity to BP230 was significantly lower compared to idiopathic BP (6, 34). It was recently reported that anti-BP180 IgG levels are often increased in patients undergoing ICI therapy, but most cases have borderline values. It cannot be excluded that ICI-induced BP results from autoAbs production against epitopes other than the non-collagenous 16A (NC16A) domain of BP180, the only one included in commercially available ELISAs (35, 38).

Interestingly, a recent study by Kramer and coworkers investigated gene expression profiling in ICI-induced BP, revealing an increased expression of PD-1, CTLA-4 and lymphocyte activation gene-3, which may be associated with favorable outcome and response to ICI therapy (34). In line with these findings, a recent study associates BP onset to improved tumor response in patients treated with anti-PD-1 therapy (31). Moreover, another paper correlates high anti-BP180 autoAbs levels with better response to ICI therapy in lung cancer patients. In this study, the autoAbs were present even before ICI initiation, suggesting they may represent a marker for BP180 overexpression in tumor tissue (39). On the other hand, eosinophilia is a marker of a longer survival in several types of cancer, including melanoma. In particular, eosinophils appear to contribute to the efficacy of immune and targeted therapy and their frequency was suggested as a predictive biomarker (40, 41). In fact, eosinophils are actively involved in carcinogenesis and modulation of the tumor microenvironment. Moreover, levels of CCL11, a chemokine responsible for eosinophil recruitment, infiltration, and degranulation, predicts eosinophil cytotoxicity and tumor response to ICI therapy (41). Specifically, eosinophils may reduce tumor burden through recruiting of cytotoxic T cells, augmenting their cytotoxic responses, and provoking cellular lysis through degranulation (41). Thus, considering the major role of eosinophils in BP pathogenesis, an additional potential mechanism at the basis of the association between melanoma and BP could be due to the proeosinophilic Th2 phenotype of ICI treated melanoma that in predisposed individuals can lead to overt BP. In this context, Hollande et al. demonstrated that ICI therapy in the presence of an inhibitor of DPP4, a chemokine modulator responsible for post-translationally cleaving of CCL11 that reduces eosinophil infiltration, enhanced the anti-tumor activities of eosinophils (42). Thus, the association between DPP4-inhibitors intake and BP onset possibly due to their ability to induce eosinophil recruitment is also in line with previous speculations.

## Expression of bullous pemphigoid antigens in tumors and melanoma

Aberrant expression of hemidesmosome-associated proteins BP230 and BP180 has been reported in different types of neoplasms, indicating their role in tumor development and invasion (43–48). Upregulation of hemidesmosome components has been reported in several SCCs (45, 49), as well as in oral dysplasia (49). Moreover, the expression of BP230 and BP180 was found in some papillary and anaplastic carcinomas and atypical adenomas, suggesting a *de novo* production and assembly of epithelial adhesion complexes antigens in thyroid malignancies (50). In a study about the transformation of oral epithelium to dysplasia and carcinoma, an augmented BP180 expression in grade II/III SCCs was found. Due to a peculiar upregulation of BP180 at the invasive front of carcinoma cells, a role in modulating carcinoma cell migration has been suggested (51). BP180 expression was also detected at the tumor-stromal interface in most basal cell carcinomas and SCCs analyzed (52). Modified expression of BP180 in SCCs resembles its behavior in colorectal cancer. Most importantly, immunohistochemical issues revealed a significant correlation between increased expression of BP180 and advanced tumor stage, lymph node and distant metastasis in colorectal cancer (53). Beyond any structural roles, BP180 is presumed to be involved in regulating cancer stem cell features like tumorigenesis and metabolic reprogramming. Hsu and colleagues further demonstrated that BP180 is required for survival and maintenance of lung cancer stem cells: the authors described that cancer stem cells increase glycolysis by the Oct4-hexokinase 2 pathway which is activated by the FAK-PI3K/AKT-GSK3β/β-catenin signaling, induced by the BP180-laminin-332 pathway (54). A recent retrospective study identified BP180 as a novel biomarker for predicting the prognosis of head and neck carcinoma (55). In particular, immunohistochemical protein evaluation of BP180 differed significantly in the presence and absence of neural invasion. As regards the laryngeal and pharyngeal cancer subgroup, a significant difference in the status of BP180 immunohistochemistry was observed according to T status (55), linking higher BP antigen expression with tumor infiltrative growth. More recently, Crespo-Bravo et al. demonstrated that circulating BP180 ectodomain levels were higher in serum of patients with different tumors, including melanoma, than in controls, suggesting a possible prognostic value of the BP antigen concentration (56).

However, other studies mentioned an opposite role of BP antigens in cancer. Downregulation of BP180 and other hemidesmosome components has been detected in prostate cancer and in the invasive cells of ductal mammary carcinoma (57, 58). As reported in more recent research, aberrant promoter methylation of *Col17A1* may predict the prognosis of patients with epithelial cancers. Its under-expression, in fact, resulted strongly related to the advanced stage, invasion, and postmenopausal status (59), while replenishing BP180 expression had an antiproliferative effect (60).

BP antigens expression in melanoma cells has also been reported. Specifically, BP180 is expressed in melanoma cells and is believed once again to play a role in promoting tumor growth and invasiveness (16). Krenacs et al. reported for the first time the accumulation of the endodomain of BP180 in malignant melanoma, but not in resting melanocytes (61). Interestingly, BP180 expression in melanomas is statistically linked to invasive tumor phenotype (62). Experiments in rodent models disclosed that truncation mutation of *Col17A1* gene promoted melanoma and supported its progression through modulation of the skin tumor microenvironment by basal keratinocytes (63). *Col17A1* is not expressed in melanocytes but in adjacent keratinocytes, dispensing a niche for melanocyte stem cells (64). Considering that BP180 is widely expressed in the brain (65), the appearance of BP180 on melanocytes during oncological transformation might be explained by the embryological origin of pigment cells. In this context, neurological diseases, including Parkinson’s disease (PD), have been associated with BP (66) and, interestingly, PD has also been associated to melanoma (67, 68). Although a clear biological explanation of these associations is still lacking, a possible link could be found in the expression of brain-derived neurotrophic factor (BDNF), that plays a pivotal role in neuronal development and synaptic plasticity. In fact, dysregulation of BDNF signaling pathway is linked to various neurodegenerative diseases, including PD (69), and serum levels of BDNF in BP sera is even lower than in PD sera (66). More interestingly, *in situ* melanoma showed a correlation with BDNF expression negativity whereas melanoma metastases were associated with BDNF immunopositivity (70) entrusting to defects in the BDNF signaling pathway the potential ability to link these three diseases. Noteworthy, *COl17A1* germline variant p.Ser1029Ala has been associated with mucosal melanoma (71). Since COl17A1 (BP180) provides a niche for melanocyte stem cells, it was hypothesized that a mutated BP180 may affect the microenvironment: in this context, this variant could form a neoantigen possibly able to induce an autoimmune response towards the hemidesmosomal autoantigen that is at the basis of BP onset.

## Human leukocyte antigen alleles in bullous pemphigoid and melanoma patients

A possible association between BP and melanoma could be hypothesized starting from genetic data. In particular, human leukocyte antigen (HLA) DQB1*03:01 allele, the HLA most associated to BP (72–75), was found to be frequently present in Caucasian patients with melanoma (76, 77), especially those with malignant melanoma, associated with a greater probability of presenting with advanced disease and experiencing tumor recurrence (77–79).

It has been proposed that this allele may serve a function in the presentation of BP antigens to autoreactive T-lymphocytes (80). Patients with the HLA-DQB1*03:01 allele show an increased T-cell avidity to several epitopes of BP180, particularly NC16A domain. Consequentially, in patients with a genetic susceptibility to BP onset upon exposure to the target antigen, T-cells activation may result in the development of functional autoimmunity against the BP180-NC16A domain, leading to clinically overt disease (81).

More HLA alleles, although less frequently, were reported to be associated to BP, such as HLA-DQA1*01:03 and HLA-DQA1*05:05 (82–84). Planelles and coworkers reported that homozygosis for HLA allele DQA1*05:05, as well as the effect of the double dosage of the DQA1*05:05‐DQB1*03:01 or DQA1*03:01‐DQB1*03:01 heterodimers, may be a potential risk factor for melanoma in the Spanish population (85). Moreover, HLA-DQA1*01:03 allele was associated with increased susceptibility to develop acral lentiginous melanoma in Mexican Mestizo patients and in the Japanese population (86, 87).

## Circulating autoantibodies to bullous pemphigoid antigens in melanoma patients

In 2010, Shimbo et al. developed a screening method to find melanoma antigens and their specific autoAbs in sera samples by *in vivo* screening of tumor-homing phages and isolation of the tumor-binding single-chain fragment variant. They showed the expression of BP230 in both melanoma cell lines and normal melanocytes but BP180, the main BP target, was not found. However, it should be considered that the commercially available BP180 ELISA is based on the NC16A domain, missing the major part of the ectodomain antigen. Interestingly, significantly higher levels of anti-BP230 autoAbs were detected in patients with both early and advanced disease stages compared to healthy volunteers, pointing to BP230 as a candidate tumor marker in melanoma (88). Attempting to reproduce these data, Gambichler and coworkers prospectively recruited 179 patients affected by cutaneous melanoma and 22 controls. Using a commercial ELISA to quantify circulating BP230, the authors failed to demonstrate any significant difference between melanoma patients at any stage and control sera (89). Noteworthy, Shimbo and coworkers did not use the cut-off value reported in the manufacturer’s instructions, redefining a new cut-off point based on the highest value measured with control sera. The authors concluded that the conflicting results might depend on the large difference in mean age between patients and healthy volunteers (62.6 and 31.6 years, respectively) enrolled by Shimbo and coworkers (89).

## Melanoma and bullous pemphigoid: a possible causative link

**Figure 1:**
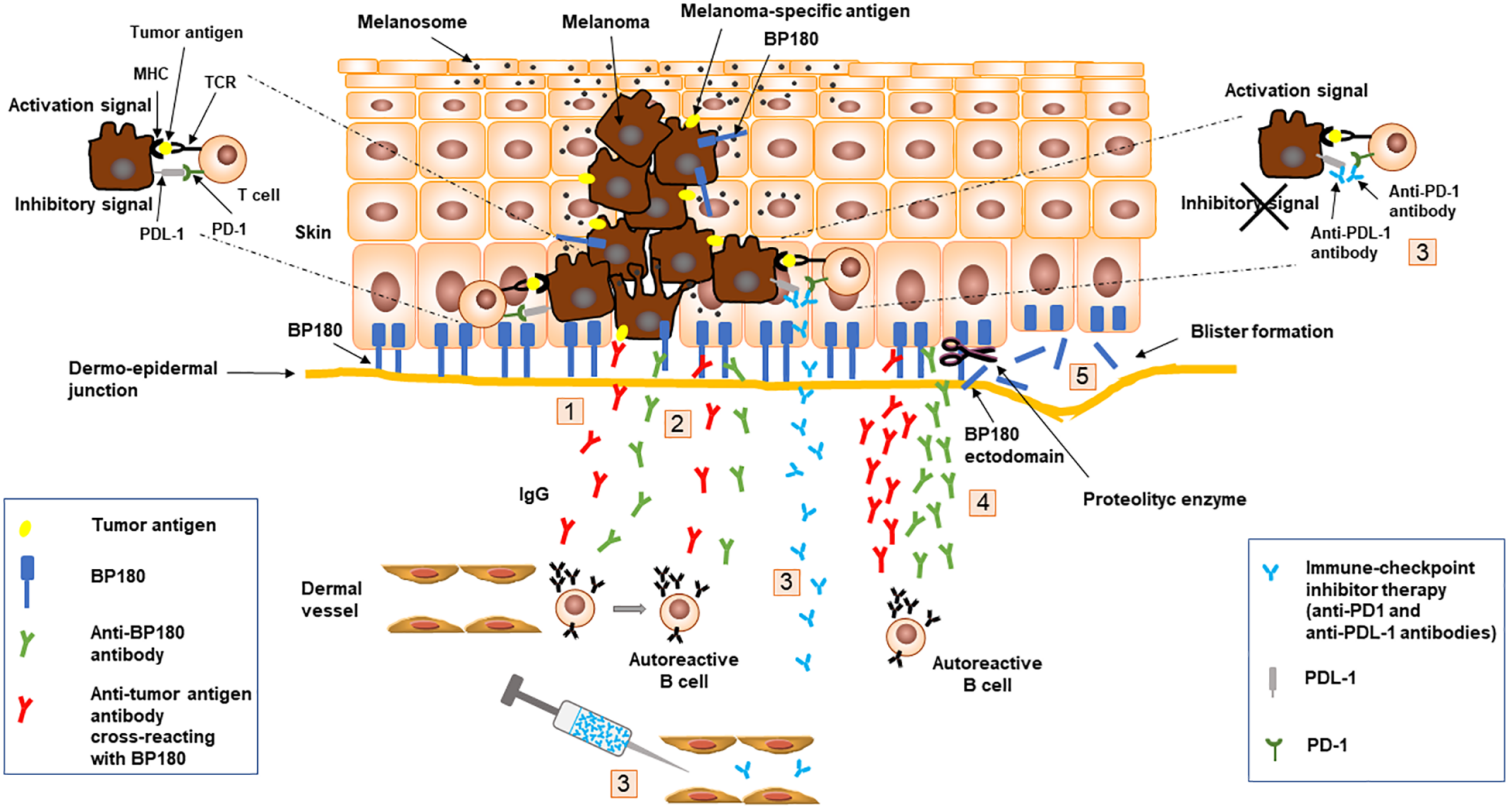
Schematic representation of speculated mechanisms at the basis of melanoma and bullous pemphigoid association. Antibodies (1) produced against tumor-specific antigens, including BP180, expressed on transformed melanocytes, might react or cross-react (2) with the basement membrane zone antigens inducing BP onset (5); Anti-PD1/PDL-1 therapy (3), aimed at blocking inhibitor signals generated by PD1 and PDL-1 interaction, might induce an immune response targeting melanoma (4) that spreads toward an autoreactive response to BP180 leading to BP onset (5). Binding of IgG to BP180 results in an inflammatory response with complement activation, degranulation of mast cells and accumulation of neutrophils and eosinophils with the release of proteolytic enzymes that cleave BP180 leading to blister formation (5). BP, bullous pemphigoid; MHC, major histocompatibility complex; PD-1, programmed cell death-1; PD-L1, programmed cell death-ligand 1; TCR, T-cell receptor.

The possible causative link between melanoma and BP is still debated and the putative pathogenetic mechanism is the result of some speculations: i) antibodies produced against tumor-specific antigens, also as a consequence of the expression of BP antigens on transformed melanocytes, might cross-react or react with the basement membrane zone antigens inducing BP onset; ii) the appearance of BP following anti-PD1/PDL-1 therapy, likely due to an immune response targeting melanoma that spreads towards an autoreactive response to BP antigens (**Figure 1**). In addition, external factors might generate both melanoma and BP in a context of a genetic predisposition such as the frequent expression of HLA-DQB1*03:01 allele.

Further studies are required to investigate the possible link between BP and melanoma and to eventually elucidate the possible mechanisms leading to the onset of this comorbidity.

## Clinical relevance

Early diagnosis of melanoma plays a crucial role for the disease outcomes, and tumor biomarkers could be fundamental to improve patient survival. In this context, the reported expression of BP180 in malignant melanoma might induce the development of circulating autoAbs, which, in turn, could be a biomarker of melanoma presence and/or an early indicator of its recurrence. Given the relevance of BP180 in cancer, the development of a liquid biopsy based on its ectodomain could represent a noninvasive diagnostic tool (56).

On the other hand, since the appearance of autoimmune response improves the outcome of cancer therapies (90), also in ICI-treated patients (31, 39), the presence of autoAbs to BP antigens could correlate with the autoimmune response to melanoma predicting the immune status of patients.

Available data and speculations discussed in the present review suggest that a specific HLA allele and the presence of autoAbs to BP antigens could be markers of a greater propensity to develop BP following ICI treatment. Accordingly, serologic testing prior to starting therapy, together with HLA characterization of melanoma patients, could highlight a higher risk of BP onset, suggesting a close monitoring to obtain an early BP diagnosis and avoid therapy cessation (39, 91). Finally, with the purpose to convert all these speculations into clinical practice, well-conducted research will be necessary.
